# Clinical thresholds in food allergy and their application in risk assessment management

**DOI:** 10.1186/2045-7022-1-S1-S65

**Published:** 2011-08-12

**Authors:** Geert Houben

**Affiliations:** 1 Business Line Food Safety TNO, Zeist, Netherlands

## 

In the EU, the use as ingredients in food of several major allergens or products derived thereof is to be labelled according to EU Directives 2003/89/EC and 2006/142/EC. This obligation only concerns the use of allergenic ingredients according to recipe. However, allergens may also be present in food due to cross contamination in food production facilities or contamination of raw materials or ingredients. Pele et al. (2007), VWA (2007) and Spanjersberg et al. (2010) have demonstrated the presence of considerable amounts of allergens in many food products that did not carry any warning for the presence of these allergens. The presence of allergens without accompanying warning obviously poses a risk to allergic consumers, as these individuals have no opportunity to judge the appropriateness of the concerning food products for them to eat (Spanjersberg et al. 2010, Sheth 2010).

In contrast to the absence of any warning on many products that do contain certain allergens, there appear to be many food products in the market that carry a precautionary (often called “may contain”) labelling to warn consumers for the possibility of unintended presence of allergens. In many cases, such precautionary labelling seems not to be based on a relevant risk but is meant as a disclaimer in case the producer cannot exclude a risk for 100%. This seems to lead to a non-selective use of this precautionary labelling, which causes other problems, such as a reduced food choice for allergic consumers and devaluation of the information value of such warning (Health Council of the Netherlands 2007). The consequence of the dilemma’s described above is that there are many products in the market with a warning while there is no or only a very small (negligible) risk and that, at the same time, there are many products without a warning that contain (sometimes very high amounts of) allergens. Precautionary labelling seems to provide the allergic consumers with no useful information anymore. Risk analysis principles can be applied to solve this problem and to bring guidance, harmonisation and transparency in information delivery regarding possible unintended presence of allergens in food products. For practical application of a risk analysis-based approach, a risk assessment methodology is essential.

We developed a risk assessment method to quantify the number of allergic consumers that may suffer allergic reactions to specific levels allergens in food products or to calculate concentration action levels that can be based on predefined tolerable risks (Spanjersberg et al. 2007 and 2010, Kruizinga 2008). Clinical threshold data are of major importance in this methodology. Clinical threshold data are available for most major food allergens for which management of cross contamination risks is needed. Suitability for use in risk assessment will be discussed and it will be demonstrated how such clinical thresholds can be used in allergen risk assessment and risk management.
